# Contribution of chronic diseases to educational disparity in disability in France: results from the cross-sectional “disability-health” survey

**DOI:** 10.1186/s13690-018-0326-9

**Published:** 2019-01-11

**Authors:** Clémence Palazzo, Renata T. C. Yokota, Jean Tafforeau, Jean-François Ravaud, Emmanuelle Cambois, Serge Poiraudeau, Herman Van Oyen, Wilma J. Nusselder

**Affiliations:** 1 Department of Public Health, Erasmus MC, Rotterdam, Netherlands; 2 Epidemiology and Public Health, Sciensano, Rue Juliette Wytsmanstraat 14, 1050 Brussels, Belgium; 3 Department of Sociology, Interface Demography, Vrije Universiteit Brussel, Brussels, Belgium; 4 INSERM, CNRS, EHESS, Université Paris Descartes, IFRH, CERMES3, Villejuif, France; 5 INED (National Institute of Demographic Studies), Paris, France; 6 INSERM, Université Sorbonne Paris Cité, Université Paris Descartes, IFRH, Centre of Research in Epidemiology and Statistics, ECaMO Team, Paris, France; 7 Department of Public Health, Ghent University, Ghent, Belgium

**Keywords:** Disability, Chronic diseases, Socioeconomic status, Educational attainment

## Abstract

**Background:**

This study aimed 1) to assess whether the contribution of chronic conditions to disability varies according to the educational attainment, 2) to disentangle the contributions of the prevalence and of the disabling impact of chronic conditions to educational disparities.

**Methods:**

Data of the 2008–09 Disability Health Survey were examined (*N* = 23,348). The disability indicator was the Global Activity Limitation Indicator (GALI). The attribution method based on an additive hazard model was used to estimate educational differences in disabling impacts and in the contributions of diseases to disability. Counterfactual analyses were used to disentangle the contribution of differences in disease prevalence vs. disabling impact.

**Results:**

In men, the main contributors to educational difference in disability prevalence were arthritis (contribution to disability prevalence: 5.7% (95% CI 5.4–6.0) for low-educated vs. 3.3% (3.0–3.9) for high-educated men), spine disorders (back/neck pain, deformity) (3.8% (3.6–4.0) vs. 1.9% (1.8–2.1)), chronic obstructive pulmonary diseases (2.4% (2.3–2.6) vs. 0.6% (0.5–0.7)) and ischemic heart /peripheral artery diseases (4.1% (3.9–4.3) vs. 2.4% (2.2–3.0)). In women, arthritis (9.5% (9.1–9.9) vs. 4.5%, (4.1–5.2)), spine disorders (4.5% (4.3–4.7) vs. 2.1% 1.9–2.3) and psychiatric diseases (3.1% (3.0–3.3) vs. 1.1% (1.0–1.3)) contributed most to education gap in disability. The educational differences were equally explained by differences in the disease prevalence and in their disabling impact.

**Conclusions:**

Public health policies aiming to reduce existing socioeconomic disparities in disability should focus on musculoskeletal, pulmonary, psychiatric and ischemic heart diseases, reducing their prevalence as well as their disabling impact in lower socioeconomic groups.

**Electronic supplementary material:**

The online version of this article (10.1186/s13690-018-0326-9) contains supplementary material, which is available to authorized users.

## Background

In recent years, there has been a growing interest in socioeconomic inequalities in disability [1, 2]. Previous studies pointed out a large educational gap in disability, in both developed [3–5] and developing countries [2, 6].

Disabling chronic diseases contribute to this gap in disability, due to unequal exposures to their risk factors [7–10] and/or due to their unequal disabling impact [3] owing to unequal recovery rates or chances to adjust to their functional consequences [11]. The few studies that assessed the contribution of chronic conditions to the educational gap in disability pointed musculoskeletal and cardiovascular diseases as the main contributors [3, 4, 12, 13]. Most of these studies [4, 13] included adults 50 years and over in developed countries. Though, studies including the younger population should be useful to better address public health strategies for three main reasons: 1) disability has increased in the young and mid-adult population in certain countries [14–17], 2) disabling diseases among younger individuals may be different from the older [15] and 3) it is necessary to prioritize the prevention and the treatment of chronic diseases in the young population to reduce their progression to disability. Another limitation of previous studies is that the disability indicator chosen for the analysis often only reflect physical functional limitations [3, 4, 12, 13], ignoring limitations due to psychiatric and neurologic disorders [15].

Another public health issue is to disentangle whether the contribution of diseases to the gap in disability is due to differences in their prevalence or in their disabling impact across education groups. Indeed, if the gap is mainly due to differences in the disabling impact of diseases [3], policies to reduce this gap should focus on the differences in the disease management, assistive devices, care giving and adaptation of environment. If the gap is mainly explained by differences in disease prevalence, focus should be on the prevention of these diseases in the most exposed groups. Within Europe, France has a low overall mortality but a high premature mortality and relatively large social gaps in health and mortality [5, 18]. The French Disability-Health Survey (DHS), a population-based, provides the opportunity to identify which diseases are important for the gap in disability prevalence [19]. The survey has been previously analyzed to assess the contribution of chronic conditions to disability and showed that neurological, musculoskeletal, and cardiovascular conditions mainly contribute to disability, and that psychiatric conditions have a heavy burden on young people [15]. However, educational differences in disability prevalence have not been studied.

This study aimed to assess whether the contribution of chronic diseases to disability varies by educational attainment in France using data from the DHS and to disentangle the contributions of differences in the prevalence and disabling impact of diseases to educational gap in disability.

## Methods

### Disability-health survey

Data from the cross-sectional population-based 2008–2009 DHS were used [19]. Our study focused on the population living in private households. The DHS methodology has been described in detail elsewhere [15, 20] and is presented in Additional file 1: Figure S1. Data were collected for 39,065 subjects across the country by face-to-face and computer-assisted personal interviewing technique conducted with the respondent (or a proxy if the respondent was not able to answer alone). The response rate was 76.6%, corresponding to 23,348 respondents aged 25 years and older. A weighting system accounted for the sampling procedure and participation to obtain representative results at the national level.

### Definition of chronic disease groups, educational level and disability

Chronic diseases were self-reported by the respondents, based on a checklist of 52 disorders. They were gathered, and 13 groups of chronic conditions were included in the study (Additional file 2: Table S1).

Education was measured according to the highest individual attainment and groups were constituted based on the international classification ISCED [21]. We considered the low-educated group (ISCED 0–1), middle-educated group (ISCED 2–4) and the high-educated group (ISCED 5–8) [22].

Disability was measured using the Global Activity Limitation Indicator (GALI). This is a single-item question on health-related activity limitations and participation restrictions [23, 24]: “ For at least the past six months, to what extent have you been limited because of a health problem in activities people usually do? Severely limited, limited but not severely and not limited at all”. People were considered as disabled if they were limited or severely limited.

### Statistical analysis

1. The prevalence of diseases and disability by education were first assessed by age-standardized weighted proportions and their 95% confidence intervals (95% CIs), using the French population age structure drawn from the 2009 population census [25].
2. The disabling impact of the 13 diseases, i.e. the disease-specific cumulative rates of disability, and their contribution to the disability prevalence were estimated using the attribution method for each education group [3, 12]. This method partitions the disability prevalence into additive contribution of chronic conditions and background, taking into account multi-morbidity; the method also account for the fact that individuals can report disability in absence of any disease (“background part”) [12]. The background can represent the disabling effect of conditions that were not included in the study, of underreported and undiagnosed conditions, of age, of other causes that were not included in the analysis [26, 27].

This method assumes that: (i) the distribution of disability by cause is entirely explained by the conditions that are reported (still present) at the time of the survey and by the background; (ii) that the diseases and background act as independent competing causes; (iii) that the cumulative rates of disability for background and diseases (in our study specific for each age group, gender and educational level) were proportionally equal in the time preceding the survey;; and (iv) the start of the time at risk for disability is the same for all diseases.

The attribution method is based on the binomial additive hazard model [27, 28] as shown in (1).

$$ {\displaystyle \begin{array}{c}{Y}_i: Bernouilli\left({\pi}_i\right)\\ {}{\pi}_i=1-\left(\exp \left(-{\eta}_i\right)\right)\\ {}{\eta}_i={\alpha}_{ae}+\sum \limits^m{\beta}_{cde}{X}_{di}\end{array}} $$

In (1), *Y*_*i*_ is the binary response variable (disability) for each individual *i*; *π*_*i*_ is the estimated probability that individual *i* is disabled; *η*_*i*_ is the overall cumulative rate of disability (linear predictor) for each individual *i*; *α*_*ae*_ is the cumulative disability rate for background by age group *a* (25–39, 40–49, 50–59, 60–69, 70–79, ≥80 years) and education *e* (low, middle, high); *β*_*cde*_ are the age- and education-specific disabling impacts of each disease *d* (1, …, m); *X*_*di*_ is the indicator variable for each disease *d* and individual *i*. To prevent an excessive number of parameters in the model, a reduced-rank regression was used [29], so the rank of the interactions was reduced to one: the age- and education-specific disabling impacts of each disease, *β*_*cde*_, were estimated as the product of one age pattern *γ*_*c*_ (25–39, 40–54, 55–69, ≥70 years) equal for each disease and a disease effect *δ*_*de*_, which was allowed to vary by disease and education, but not by age. In our analysis, model (1) was fitted for men and women separately. The respective contribution of chronic conditions and background to the disability prevalence could be calculated as explained by Yokota et al. [30].

Sampling weights were used to calculate the prevalence of disability and diseases, the disabling impacts and the contributions of diseases to disability. Bootstrapping with 1000 replicates was used to obtain confidence intervals for the disabling impacts and the contributions of chronic conditions [31].

(3) We assessed the contribution of prevalence vs disabling impact diseases to the educational gap in disability as follows. First, we calculated the age-standardized contribution of the disease in the low-educated group minus the age-standardized contribution in the high-educated group (baseline situation). Second we used two counterfactual scenarios: in the first one, we applied the age-standardized disease prevalence of the high-educated group to the low-educated group and assessed the “counterfactual” educational gap in disability, which provides this time the contribution of the educational differences in the disabling impact of diseases (being the remaining gap if the prevalence of diseases was equal); in the second scenario, we applied the disabling impacts of the diseases of the high-educated group to the low-educated group and assessed the “counterfactual” educational gap in disability, which provides the contribution of the educational difference in the diseases prevalence (being the remaining gap if the disabling impact of diseases was equal).

Statistical analyses were carried out in SAS 9.2 (SAS Inst., Cary, NC) and R version 3.3.2. To fit the binomial additive hazard model and estimate the contributions, the software developed by Nusselder and Looman (2010) [27] was used and is available upon request to the authors of the method (w.nusselder@erasmusmc.nl).

## Results

1. Prevalence of disability by education

**Fig. 1:**
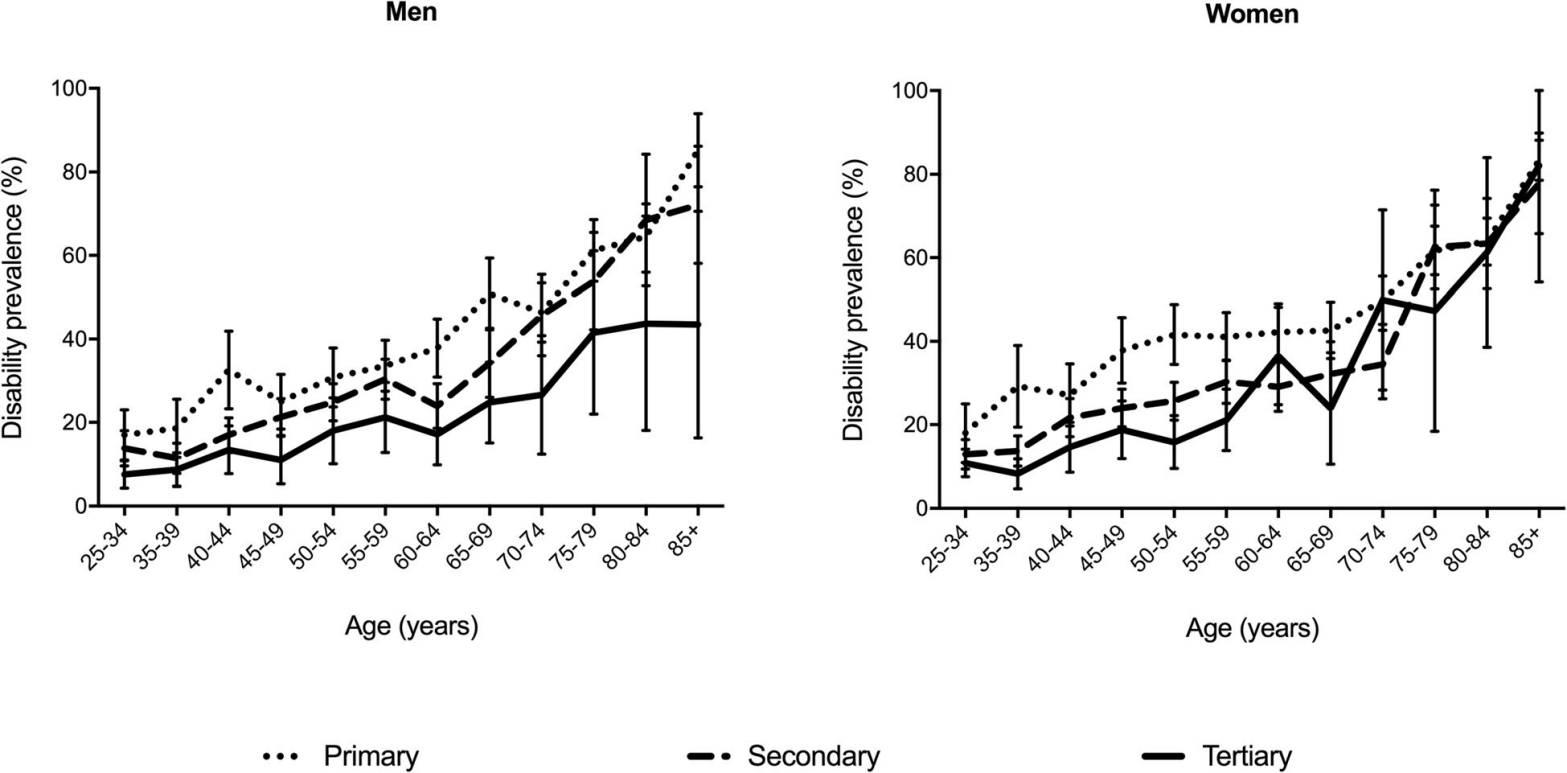
Prevalence of disability according to age groups and educational level for men and women

We observed a clear educational gradient in disability (Fig. 1) with higher disability among lower educated. The Odds Ratio of being disabled when having a primary versus a tertiary educational level was 4.74 (95% CI: 3.99–5.62) for women and 4.24 (3.51–5.14) for men. The absolute differences between high- and low-educated men varied from 9.5% in the 25–34 age group up to 41.8% in the 85+ age group (with large confidence intervals). For women, the differences were smaller than for men, but they were the largest at mid-adult ages and converged at older ages, varying from 25.8% for the 50–54 age group to 1.3% for the 85+ age group to.

(2) Prevalence and disabling impact of chronic diseases according to the educational level

Table 1 provides the sex-specific age-standardized prevalence of diseases and their disabling impact in the three education groups. For men, chronic diseases were all more prevalent in the low-educated group. The largest difference was observed for musculoskeletal diseases, both for spine disorders (21.35% in low-educated men vs 15.78% in high-educated men) and arthritis (21.05% vs 11.99%), and for diabetes (10.69% vs 5.15%) (no overlapping of confidence intervals). For women, almost all chronic diseases were more prevalent in the low-educated group. The largest differences were observed for arthritis (29.95% vs 21.03%), diabetes (8.43% vs 2.19%) and psychiatric diseases (12.92% vs 7.90%) (no overlapping of confidence intervals).

**Table 1:** Age-standardized prevalence and disabling impact of chronic conditions by educational level and gender, the Disability-Health survey, France, 2008–2009 (*N* = 23,348 respondents 25 years and older)

	Primary		Secondary		Tertiary	
	Prevalence^a^, % (95% CI)	Disabling impact^b^, mean (95% CI)	Prevalence^a^, % (95% CI)	Disabling impact^b^, mean (95% CI)	Prevalence^a^, % (95% CI)	Disabling impact^b^, (mean, 95% CI)
Men						
Spine disorders	21.35 (20.23–22.52)	0.28 (0.19–0.36)	18.47 (17.46–19.53)	0.18 (0.13–0.23)	15.78 (14.14–17.57)	0.15 (0.09–0.21)
Arthritis	21.05 (19.99–22.15)	0.46 (0.35–0.57)	17.71 (16.67–18.79)	0.53 (0.42–0.63)	11.99 (10.53–13.62)	0.38 (0.24–0.52)
Dementia	1.51 (1.29–1.75)	0.89 (0.25–1.53)	1.55 (1.23–1.96)	0.62 (−0.01–1.25)	1.30 (0.87–1.95)	3.28 (− 0.56–7.12)
Stroke	2.61 (2.31–2.95)	0.85 (0.37–1.32)	2.46 (2.12–2.86)	0.76 (0.34–1.17)	1.79 (1.32–2.43)	0.41 (− 0.07–0.89)
Other neurologic diseases	2.15 (1.88–2.45)	0.99 (0.47–1.50)	2.04 (1.76–2.36)	1.08 (0.61–1.55)	1.32 (1.02–1.70)	0.47 (0.10–0.85)
Ischemic heart diseases/PAD	9.93 (9.22–10.69)	0.83 (0.62–1.05)	9.37 (8.56–10.26)	0.57 (0.38–0.76)	6.05 (4.99–7.32)	0.64 (0.32–0.96)
Other heart diseases	6.28 (5.75–6.85)	0.61 (0.33–0.88)	6.38 (5.74–7.07)	0.29 (0.11–0.46)	5.65 (4.54–7.01)	0.31 (0.09–0.53)
COPD	10.10 (9.37–10.89)	0.42 (0.27–0.57)	7.67 (6.98–8.43)	0.28 (0.17–0.38)	5.51 (4.55–6.65)	0.14 (0.02–0.26)
Psychiatric diseases	7.79 (7.18–8.45)	0.65 (0.43–0.87)	4.85 (4.42–5.33)	0.54 (0.38–0.71)	5.02 (4.24–5.92)	0.36 (0.17–0.54)
Sensorial diseases	0.96 (0.76–1.20)	0.46 (−0.03–0.96)	1.49 (1.15–1.92)	0.18 (−0.11–0.47)	0.45 (0.29–0.71)	0.09 (− 0.19–0.38)
Cancer	3.37 (2.97–3.82)	0.66 (0.37–0.95)	3.11 (2.64–3.66)	0.38 (0.15–0.61)	1.92 (1.45–2.56)	0.49 (0.12–0.85)
Diabetes	10.69 (9.95–11.47)	0.20 (0.09–0.31)	7.23 (6.56–7.97)	0.13 (0.04–0.23)	5.15 (4.28–6.18)	0.16 (0.01–0.30)
Accidents	5.37 (4.84–5.94)	0.96 (0.62–1.29)	4.59 (4.16–5.07)	0.61 (0.44–0.78)	3.29 (2.64–4.10)	0.45 (0.22–0.69)
Women						
Spine disorders	23.00 (22.10–23.93)	0.33 (0.25–0.41)	23.08 (21.99–24.21)	0.30 (0.24–0.35)	22.32 (20.54–24.21)	0.13 (0.08–0.18)
Arthritis	29.95 (28.95–30.97)	0.55 (0.47–0.64)	23.77 (22.65–24.93)	0.43 (0.36–0.51)	21.03 (19.14–23.06)	0.34 (0.23–0.45)
Dementia	1.31 (1.16–1.47)	1.44 (0.79–2.09)	0.82 (0.65–1.04)	1.22 (0.24–2.20)	0.53 (0.29–0.96)	3.00 (−3.34–9.35)
Stroke	1.91 (1.72–2.13)	0.79 (0.35–1.22)	1.37 (1.12–1.68)	1.76 (0.72–2.80)	1.74 (1.22–2.46)	1.15 (0.07–2.23)
Other neurologic diseases	2.12 (1.88–2.39)	1.23 (0.66–1.79)	1.45 (1.25–1.69)	1.20 (0.67–1.72)	1.66 (1.31–2.10)	0.79 (0.35–1.23)
Ischemic heart diseases/PAD	3.74 (3.45–4.06)	0.94 (0.61–1.27)	3.22 (2.78–3.73)	0.80 (0.41–1.20)	3.44 (2.49–4.75)	0.34 (−0.16–0.83)
Other heart diseases	6.16 (5.74–6.59)	0.75 (0.53–0.97)	4.82 (4.32–5.37)	0.34 (0.15–0.53)	6.94 (5.64–8.51)	0.48 (0.16–0.81)
COPD	8.15 (7.61–8.71)	0.22 (0.10–0.33)	7.44 (6.81–8.12)	0.35 (0.25–0.45)	7.36 (6.20–8.72)	0.35 (0.22–0.48)
Psychiatric diseases	12.92 (12.25–13.61)	0.44 (0.32–0.57)	9.09 (8.49–9.73)	0.34 (0.24–0.44)	7.90 (6.92–9.00)	0.23 (0.11–0.34)
Sensorial diseases	0.56 (0.45–0.69)	1.14 (0.31–1.96)	0.39 (0.29–0.52)	0.84 (0.04–1.64)	0.65 (0.43–0.99)	0.50 (−0.27–1.28)
Cancer	2.98 (2.65–3.35)	0.90 (0.59–1.22)	2.49 (2.15–2.88)	1.08 (0.72–1.44)	3.72 (2.83–4.86)	1.46 (0.82–2.10)
Diabetes	8.43 (7.94–8.95)	0.38 (0.25–0.51)	4.79 (4.28–5.34)	0.29 (0.15–0.44)	2.19 (1.64–2.93)	0.43 (0.03–0.83)
Accidents	3.22 (2.89–3.60)	0.28 (0.06–0.50)	3.30 (2.92–3.73)	0.53 (0.31–0.74)	3.85 (3.12–4.74)	0.93 (0.56–1.31)

*CI* Confidence interval; Prevalence rates are weighted and age-standardized to the French population of men and women estimated by the 2009 census (French National Institute of Statistics and Economic Studies). The disabling impact is the disease-specific cumulative rates of disability for each disease (that is the β in the linear predictor presented in formula (1)). Spine disorders (low back pain, neck pain, spine deformity), arthritis (rheumatoid arthritis, other type of inflammatory arthritis, osteoarthritis), Parkinson and Alzheimer diseases (and other types of dementia), neurologic diseases (epilepsy, multiple sclerosis), stroke, ischemic heart disease/peripheral artery disease (PAD), other heart diseases (arrhythmia, heart failure), chronic obstructive pulmonary diseases (COPD) (asthma, chronic bronchitis), psychiatric diseases (anxiety, depression, schizophrenia and autism), sensorial diseases (blindness or severe visually impairment, deafness or serious hearing loss), cancer, diabetes, and sequelae of injury

Most diseases had a higher disabling impact in the low-educated group. For men, the largest difference was observed for neurologic diseases (0.99 in low-educated men vs 0.47 in high-educated men) and accidents (0.96 vs 0.45). For women, the difference in disabling impact was largest for neurologic diseases (1.23 vs 0.79) and sensory impairment (1.14 vs 0.50). Dementia might have a higher impact in the high-educated than in low-educated group (3.28 vs 0.89 for men and 3.00 vs 1.44 for women), but there was an overlapping of confidence intervals.

**Fig. 2:**
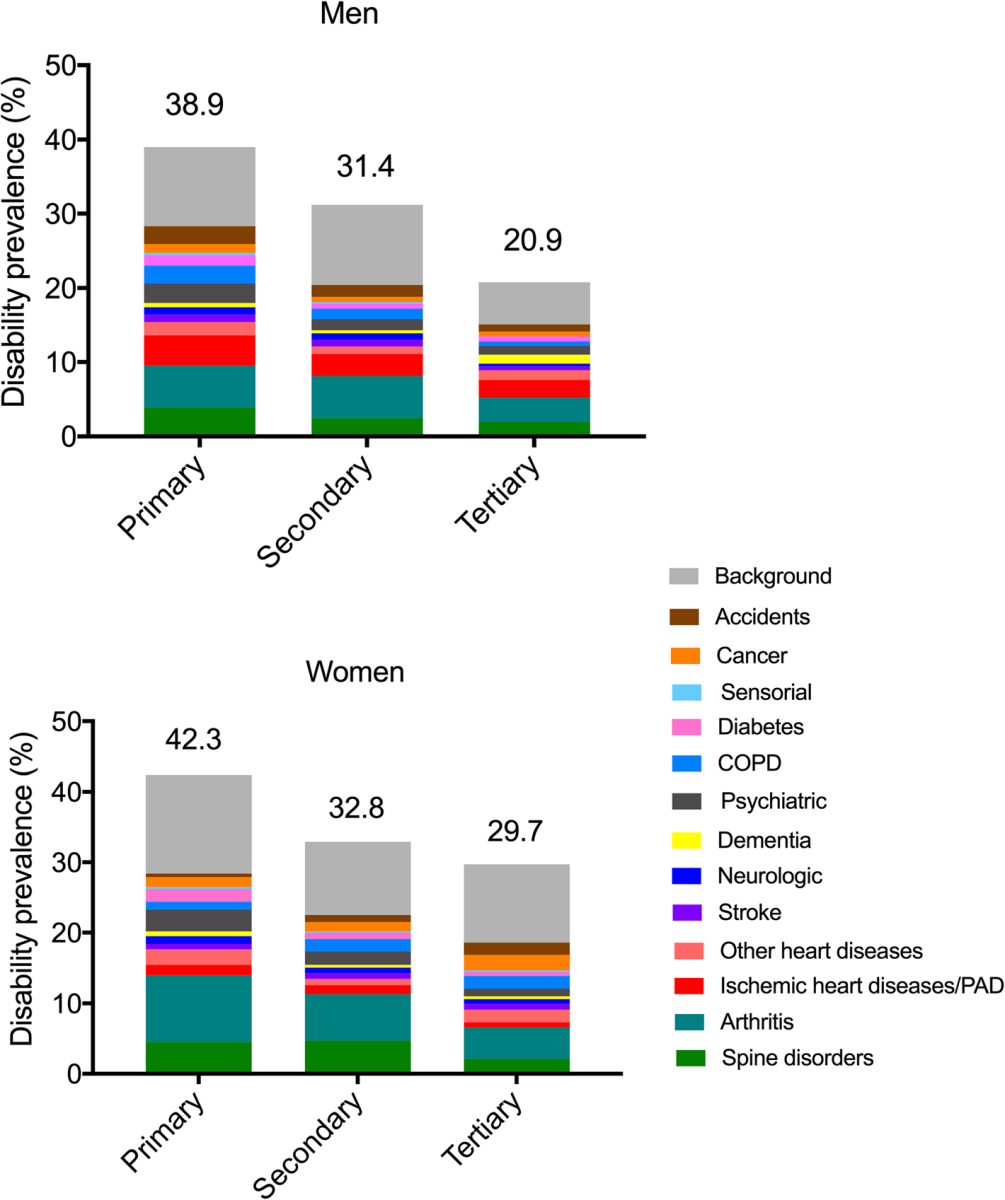
Age-standardized absolute contribution of chronic conditions and background to the prevalence of disability according to educational level

Table 2 and Fig. 2 present the contribution of the diseases to the disability prevalence in the low- and high-educated groups (see also Additional file 3: Table S2 for the value in the middle-educated group). Arthritis, ischemic heart disease/ peripheral artery disease (PAD), and spine disorders were the main contributors to disability for all education groups. The largest differences in percentage points between low- and high-educated men were observed for arthritis (2.4 percentage points), spine disorders (1.9), chronic obstructive pulmonary diseases (COPD) (1.8) and ischemic heart disease/PAD (1.7). Only dementia showed a larger contribution in the high-educated group in men, in relation to its larger disabling impact above mentioned. For women, arthritis and spine disorders contributed most to the disability prevalence in all educational level groups. The largest differences between low- and high-educated women were observed for arthritis, spine disorders, and psychiatric diseases (5.0, 2.4 and 2.0 percentages point differences, respectively). Accidents, cancer and COPD had a greater impact in the high- vs low-educated women. There was an educational gradient in the part of disability attributable to background; background contributed more for lower educated (5.0 percentage point difference between low- and high-educated men and 2.9 between low- and high-educated women).

(3) Contribution of differentiated prevalence vs. disabling impact of disease to the educational gap in disability

**Table 2:** Absolute contributions of diseases to educational disparity in disability prevalence in baseline situation and under counterfactual scenarios, the Disability Health Survey, France, 2008–2009. Results are % (95% CI)

	Contributions of Diseases to Total Prevalence of Disability Among Groups with a Tertiary and Elementary Education, Percentage Points				Difference Between Elementary and Tertiary Education Representing Contribution to Disability Disparity, Percentage Points		
	Primary (1)	Tertiary (2)	Primary, counterfactual with disease prevalence of tertiary (3)	Primary, counterfactual with disabling Impact of Tertiary (4)	Baseline Situation (No Counterfactual) (1–2)	Counterfactual With Disease Prevalence of primary Equal to Tertiary (3–2) ^a^	Counterfactual with disabling Impact of primary equal to tertiary (4–2) ^b^
Men							
Spine disorders	3.8 (3.6–4.0)	1.9 (1.8–2.1)	3.2	2.3	1.9	1.3	0.4
Arthritis	5.7 (5.4–6.0)	3.3 (3.0–3.9)	3.4	5.3	2.4	0.1	2.0
Dementia	0.6 (0.6–0.6)	1.2 (0.9–1.6)	0.5	1.2	−0.6	− 0.7	0.0
Stroke	1.0 (0.9–1.0)	0.5 (0.5–0.6)	0.7	0.6	0.5	0.2	0.1
Neurologic diseases	1.0 (0.9–1.0)	0.4 (0.4–0.4)	0.7	0.6	0.6	0.3	0.2
Ischemic heart diseases/PAD	4.1 (3.9–4.3)	2.4 (2.2–3.0)	2.8	3.7	1.7	0.4	1.3
Other heart diseases	1.8 (1.7–1.9)	1.3 (1.1–1.7)	2.0	1.2	0.5	0.7	−0.1
COPD	2.4 (2.3–2.6)	0.6 (0.5–0.7)	1.5	1.0	1.8	0.9	0.4
Psychiatric diseases	2.6 (2.5–2.8)	1.2 (1.1–1.3)	1.6	1.8	1.4	0.4	0.6
Sensorial diseases	0.3 (0.3–0.3)	0.0 (0.0–0.0)	0.1	0.1	0.3	0.1	0.1
Cancer	1.2 (1.1–1.2)	0.6 (0.6–0.8)	0.7	1.0	0.6	0.1	0.4
Diabetes	1.4 (1.4–1.5)	0.7 (0.7–0.8)	0.9	1.2	0.7	0.2	0.5
Accidents	2.4 (2.3–2.5)	1.0 (0.9–1.1)	1.2	1.5	1.4	0.2	0.5
Background	10.7 (10.2–11.3)	5.7 (5.3–6.4)	11.6	11.4	5.0	5.9	5.7
Disability prevalence	38.9 (37.3–41.0)	20.9 (19.2–24.0)	30.8	32.8	18	9.9	11.9
Women							
Spine disorders	4.5 (4.3–4.7)	2.1 (1.9–2.3)	4.5	2.0	2.4	2.4	−0.1
Arthritis	9.5 (9.1–9.9)	4.5 (4.1–5.2)	7.0	6.6	5.0	2.5	2.1
Dementia	0.7 (0.7–0.8)	0.4 (0.3–0.5)	0.5	1.0	0.3	0.1	0.6
Stroke	0.7 (0.6–0.7)	0.8 (0.7–1.1)	0.8	1.2	−0.1	0.0	0.4
Neurologic diseases	1.1 (1.0–1.2)	0.7 (0.7–0.8)	0.8	0.9	0.4	0.1	0.2
Ischemic heart diseases/PAD	1.5 (1.4–1.6)	0.7 (0.6–0.8)	1.5	0.7	0.8	0.8	0.0
Other heart diseases	2.2 (2.1–2.3)	1.8 (1.6–2.3)	2.9	1.7	0.4	1.1	−0.1
COPD	1.1 (1.0–1.1)	1.8 (1.6–2.1)	1.0	1.8	−0.7	−0.8	0.0
Psychiatric diseases	3.1 (3.0–3.3)	1.1 (1.0–1.3)	1.9	1.9	2.0	0.8	0.8
Sensorial diseases	0.3 (0.3–0.3)	0.2 (0.2–0.2)	0.2	0.2	0.1	0.0	0.0
Cancer	1.4 (1.3–1.5)	2.2 (2.0–2.6)	1.5	1.9	−0.8	−0.7	−0.3
Diabetes	1.8 (1.8–1.9)	0.6 (0.5–0.7)	0.4	2.2	1.2	−0.2	1.6
Accidents	0.5 (0.5–0.6)	1.7 (1.5–1.9)	0.8	1.5	−1.2	−0.9	− 0.2
Background	14.0 (13.5–14.6)	11.1 (9.7–13.9)	14.3	14.7	2.9	3.2	3.6
Disability prevalence	42.3 (40.9–44.1)	29.7 (26.7–35.2)	38.0	37.8	12.6	8.3	8.1

^a^This represents the part of educational disparity explained by the difference of disabling impact of diseases between the primary and the tertiary educational level group

^b^This represents the part of educational disparity explained by the difference of diseases prevalence between the primary and the tertiary educational level group

*PAD* Peripheral Artery Diseases, *COPD* Chronic Obstructive Pulmonary Diseases

Contributions were age-standardized to the French population of men and women estimated by the 2009 census (French National Institute of Statistics and Economic Studies). There were 23,348 respondents 25 years and older

The first counterfactual scenario (Table 2) applied the prevalence of diseases of the high-educated men to the low-educated men, keeping their disabling impact: the remaining disability gap, due to difference in disabling impact, was 9.9% instead of 18% in baseline situation in men and 8.3% instead of 12.6% in women.

In the second scenario using the disabling impact of diseases of the high-educated group, the gap between the low- and the high-educated individuals, due to the difference in the prevalence of the diseases, was 11.9% instead of 18% in baseline situation in men and 8.1% instead of 12.6% in women.

For women, the differences in the prevalence and in the disabling impact contribute to the same extent to the overall disability prevalence, while for men the differences in the prevalence of the diseases contribute slightly more than the differences in disabling impact.

## Discussion

This study shows a clear educational gradient in disability in both men and women, as well as differentials in the contribution of self-reported chronic diseases to disability in France, using data representative of the household population. In men, the main contributors to educational gap in disability were arthritis, spine disorders, COPD and ischemic heart disease/PAD. In women, arthritis, spine disorders, psychiatric diseases and ischemic heart disease/PAD contributed most.

Compared to previous works, our study considered accidents and psychiatric disorders: we found that psychiatric disorders were the third contributor to the educational gap in disability in women and the fifth in men, confirming the importance of these conditions to social differences, in addition to musculoskeletal disorders [32–34]. Apart from these considerations, our results were close to other studies findings. In the Dutch population, Klijs et al. found that musculoskeletal conditions, PAD and lung diseases contributed most to the disability gap, using the same statistical method [35]. Psychiatric diseases were not included, but a sensitivity analysis also suggested a substantial contribution of mental illness to the disability gap. In the Belgian population, arthritis, back complaints and COPD mainly contributed to educational gap in disability-free life expectancy [12]. In the Finnish population, musculoskeletal and cardiovascular diseases mainly explained the educational gap in mobility restriction [4].

Using counterfactual scenario, we found that the educational gap in disability was equally explained by the educational differences in the prevalence and in the disabling impact of diseases in women; slightly more by the differences in prevalence of diseases in men. Klijs et al. found that differences in the disabling impact of diseases were more important than differences in their prevalence in the educational gap in the Dutch study [3]. This may be due to differences in disability definition, diseases, study population, survey methodology, as well as in public health policies between France and the Netherlands. These results suggest that in France educational gap in disability could be reduced both by reducing the differentials in the diseases prevalence and in their disabling impact. For the latter, policies could focus on improving diseases management and promoting coping strategies for their functional consequences (adjustment of activities, adaptation of the environment and public transportation, accessibility of assistive devices), especially in the low-educated group who seem to benefit less. The reduction of the educational gap in disability could also results prevention strategies towards the diseases that contribute the most to disability, focusing on the low-educated groups. For instance, arthritis could be reduced by improving detrimental work exposures, more frequent in low skilled jobs, encouraging physical activity or preventing overweight which are also linked to the social status; COPD by preventing tobacco smoking; accidents by protecting the most exposed workers or promoting safe driving practice.

The outcomes of the counterfactual scenarios may for some diseases seem inconsistent with the age-standardized prevalence and disabling impact of diseases presented in Table 1. For instance, the age-standardized prevalence of “other heart diseases” is slightly lower among high-educated men (5.7%) than low-educated men (6.3%), but the contribution of this disease to disability prevalence is 2.0% in the counterfactual which replaces the prevalence of the low educated by that of the high educated and 1.8% in the baseline situation. This may be explained by the fact that the age-standardized prevalence masks age-specific variations; in this case, a higher prevalence of “other heart diseases” in oldest high-educated group (28.8%) than low-educated group (15.1%). Excluding the 80+ age group from the analyses, the contribution is 1.2% in the counterfactual and 1.3% the in baseline situation.

This study has some limitations. Firstly, there might be an educational difference in reporting diseases: wealthier and more educated individuals tend to have a greater knowledge about disease and an earlier diagnosis due to better health care access [36]. This reporting bias may underestimate the prevalence of diseases among low-educated individuals. Furthermore, social desirability bias may be larger in low-educated than in high-educated individuals, what can for instance impact the reporting of driving behaviors and minimize the contribution of accidents [37]. Also, severe chronic conditions might be under-represented in surveys like HSM because frail people are less able to be contacted and to participate in surveys, and a proxy respondent is not always present. This may differ between educational groups and may partly explain the higher disabling impact and contribution of dementia if low educated persons with dementia are less represented in the survey. Another possible explanation is that the high-educated men may have intellectually demanding activities/jobs that help detecting cognitive disorders at earlier stages. Another limitation is that we cannot draw any causality link as this is a cross-sectional study. On the one hand diseases can result from a fragile status induced by low-education, such as unskilled job exposures [38]; on the other hand, teenagers with a disabling disease may not be able to complete a secondary education which would also contribute to the association between low-education and disability [39, 40]. However, there are many evidences that life and work conditions associated with low education might expose to the risk of disabling diseases. Finally, our study is based on three educational groups which are heterogeneous: low-education in young and old generations reflect substantially different socioeconomic situation.

## Conclusion

This study indicates important educational inequalities in disability prevalence, with large contributions of musculoskeletal, pulmonary and psychiatric diseases both through higher disease prevalence and disabling impact among the lower educated. Public health policies aiming to reduce existing socioeconomic disparities in disability in France should focus on these diseases, addressing both primary prevention reducing their prevalence and targeting persons with the diseases, reducing the consequences of these diseases in daily life.

## Additional files

Additional file 1: **Figure S1.** Design of the representative national “Disability-Health” survey. INSEE = French National Institute of Statistics and Economic Studies. (TIFF 2414 kb)

Additional file 2: **Table S1.** Detailed description of the diseases included in each group of chronic conditions. (DOCX 66 kb)

Additional file 3: **Table S2.** Age-standardized absolute contributions of diseases to disability prevalence according to the educational attainment, the Disability Health Survey, France, 2008–2009 (*N* = 23,348 respondents 25 years and older). Results are % (95% CI) (DOCX 97 kb)

## Abbreviations

CI: Confidence Interval
COPD: Chronic Obstructive Pulmonary Diseases
DHS: Disability-Health Survey
GALI: Global Activity Limitation Indicator
ISCED: International Standard Classification of EDucation
PAD: Peripheral Artery Disease
