# Supplementary figures and images for: Contribution of chronic diseases to educational disparity in disability in France: results from the cross-sectional “disability-health” survey
Source: Arch Public Health. 2019 Jan 11;77:2. doi: 10.1186/s13690-018-0326-9 (PMC6330417; doi:10.1186/s13690-018-0326-9)

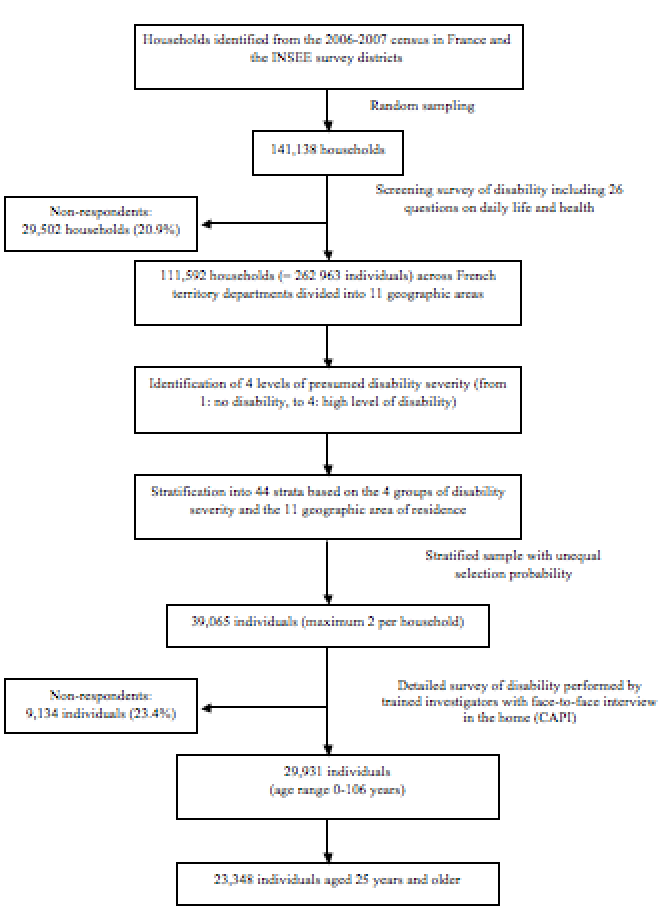

Supplement: Supplementary file 1 — Figure S1. Design of the representative national “Disability-Health” survey. INSEE = French National Institute of Statistics and Economic Studies. (TIFF 2414 kb) [file 13690_2018_326_MOESM1_ESM.tiff]
