# Supplementary material for: Contribution of chronic diseases to educational disparity in disability in France: results from the cross-sectional “disability-health” survey
Source: Arch Public Health. 2019 Jan 11;77:2. doi: 10.1186/s13690-018-0326-9 (PMC6330417; doi:10.1186/s13690-018-0326-9)
Supplement: Supplementary file 2 — Table S1. Detailed description of the diseases included in each group of chronic conditions. (DOCX 66 kb) [file 13690_2018_326_MOESM2_ESM.docx]

| **Chronic conditions** | **Included diseases** |
| --- | --- |
| Spine disorders | Low back pain, neck pain, spine deformity |
| Arthritis | Rheumatoid arthritis, other type of inflammatory arthritis, osteoarthritis |
| Dementia | Parkinson and Alzheimer diseases and other types of dementia |
| Neurologic diseases | Epilepsy, multiple sclerosis |
| Stroke | Stroke |
| Ischemic heart disease/peripheral artery disease (PAD) | Ischemic heart disease/peripheral artery disease |
| Other heart diseases | Arrhythmia, heart failure |
| Chronic obstructive pulmonary diseases (COPD) | Asthma, chronic bronchitis |
| Psychiatric diseases | Anxiety, depression, schizophrenia and autism |
| Sensorial impairment | Blindness or severe visually impairment, deafness or serious hearing loss |
| Cancer | Cancer |
| Diabetes | Diabetes |
| Accidents | Sequelae of injury |

**Table S1**. Detailed description of the diseases included in each group of chronic conditions
