# Supplementary material for: Contribution of chronic diseases to educational disparity in disability in France: results from the cross-sectional “disability-health” survey
Source: Arch Public Health. 2019 Jan 11;77:2. doi: 10.1186/s13690-018-0326-9 (PMC6330417; doi:10.1186/s13690-018-0326-9)
Supplement: Supplementary file 3 — Table S2. Age-standardized absolute contributions of diseases to disability prevalence according to the educational attainment, the Disability Health Survey, France, 2008–2009 (N = 23,348 respondents 25 years and older). Results are % (95% CI) (DOCX 97 kb) [file 13690_2018_326_MOESM3_ESM.docx]

|  | **Primary** | **Secondary** | **Tertiary** |
| --- | --- | --- | --- |
| **Men** |  |  |  |
| Spine disorders | 3.8 (3.6-4.0) | 2.4 (2.3-2.5) | 1.9 (1.8-2.1) |
| Arthritis | 5.7 (5.4-6.0) | 5.7 (5.3-6.1) | 3.3 (3.0-3.9) |
| Dementia | 0.6 (0.6-0.6) | 0.4 (0.4-0.5) | 1.2 (0.9-1.6) |
| Stroke | 1.0 (0.9-1.0) | 0.9 (0.8-1.0) | 0.5 (0.5-0.6) |
| Neurologic diseases | 1.0 (0.9-1.0) | 0.9 (0.9-1.0) | 0.4 (0.4-0.4) |
| Ischemic heart diseases/PAD | 4.1 (3.9-4.3) | 3.0 (2.8-3.4) | 2.4 (2.2-3.0) |
| Other heart diseases | 1.8 (1.7-1.9) | 1.0 (1.0-1.2) | 1.3 (1.1-1.7) |
| COPD | 2.4 (2.3-2.6) | 1.4 (1.3-1.5) | 0.6 (0.5-0.7) |
| Psychiatric diseases | 2.6 (2.5-2.8) | 1.5 (1.5-1.6) | 1.2 (1.1-1.3) |
| Sensorial diseases | 0.3 (0.3-0.3) | 0.2 (0.2-0.2) | 0.0 (0.0-0.0) |
| Cancer | 1.2 (1.1-1.2) | 0.7 (0.7-0.8) | 0.6 (0.6-0.8) |
| Diabetes | 1.4 (1.4-1.5) | 0.7 (0.7-0.8) | 0.7 (0.7-0.8) |
| Accidents | 2.4 (2.3-2.5) | 1.6 (1.6-1.7) | 1.0 (0.9-1.1) |
| Background | 10.7 (10.2-11.3) | 10.8 (10.0-11.8) | 5.7 (5.3-6.4) |
| Disability prevalence | 38.9 (37.3-41.0) | 31.4 (29.5-34.1) | 20.9 (19.2-24.0) |
| **Women** |  |  |  |
| Spine disorders | 4.5 (4.3-4.7) | 4.7 (4.5-4.9) | 2.1 (1.9-2.3) |
| Arthritis | 9.5 (9.1-9.9) | 6.6 (6.3-6.9) | 4.5 (4.1-5.2) |
| Dementia | 0.7 (0.7-0.8) | 0.4 (0.4-0.5) | 0.4 (0.3-0.5) |
| Stroke | 0.7 (0.6-0.7) | 0.8 (0.8-0.9) | 0.8 (0.7-1.1) |
| Neurologic diseases | 1.1 (1.0-1.2) | 0.8 (0.7-0.8) | 0.7 (0.7-0.8) |
| Ischemic heart diseases/PAD | 1.5 (1.4-1.6) | 1.3 (1.2-1.4) | 0.7 (0.6-0.8) |
| Other heart diseases | 2.2 (2.1-2.3) | 0.9 (0.9-1.0) | 1.8 (1.6-2.3) |
| COPD | 1.1 (1.0-1.1) | 1.7 (1.6-1.8) | 1.8 (1.6-2.1) |
| Psychiatric diseases | 3.1 (3.0-3.3) | 1.9 (1.8-2.0) | 1.1 (1.0-1.3) |
| Sensorial diseases | 0.3 (0.3-0.3) | 0.2 (0.2-0.2) | 0.2 (0.2-0.2) |
| Cancer | 1.4 (1.3-1.5) | 1.3 (1.2-1.4) | 2.2 (2.0-2.6) |
| Diabetes | 1.8 (1.8-1.9) | 0.9 (0.8-0.9) | 0.6 (0.5-0.7) |
| Accidents | 0.5 (0.5-0.6) | 1.0 (1.0-1.0) | 1.7 (1.5-1.9) |
| Background | 14.0 (13.5-14.6) | 10.4 (9.7-11.2) | 11.1 (9.7-13.9) |
| Disability prevalence | 42.3 (40.9-44.1) | 32.8 (31.2-34.7) | 29.7 (26.7-35.2) |

**Table S2**. Age-standardized absolute contributions of diseases to disability prevalence according to the educational attainment, the Disability Health Survey, France, 2008-2009 (N= 23,348 respondents 25 years and older). Results are % (95% CI)

PAD: Peripheral Artery Diseases

COPD: Chronic Obstructive Pulmonary Diseases
